# UISTD: A Trust-Aware Model for Diverse Item Personalization in Social Sensing with Lower Privacy Intrusion

**DOI:** 10.3390/s18124383

**Published:** 2018-12-11

**Authors:** Hongchen Wu, Mingyang Li, Huaxiang Zhang

**Affiliations:** School of Information Science and Engineering, Shandong Normal University, Jinan 250014, China; a546941566@163.com (M.L.); huaxzhang@sdnu.edu.cn (H.Z.)

**Keywords:** privacy intrusion, social sensing, trust relationship, *k*-means clustering, core user, information disclosure

## Abstract

Privacy intrusion has become a major bottleneck for current trust-aware social sensing, since online social media allows anybody to largely disclose their personal information due to the proliferation of the Internet of Things (IoT). State-of-the-art social sensing still suffers from severe privacy threats since it collects users’ personal data and disclosure behaviors, which could raise user privacy concerns due to data integration for personalization. In this paper, we propose a trust-aware model, called the User and Item Similarity Model with Trust in Diverse Kinds (UISTD), to enhance the personalization of social sensing while reducing users’ privacy concerns. UISTD utilizes user-to-user similarities and item-to-item similarities to generate multiple kinds of personalized items with common tags. UISTD also applies a modified *k*-means clustering algorithm to select the core users among trust relationships, and the core users’ preferences and disclosure behaviors will be regarded as the predicted disclosure pattern. The experimental results on three real-world data sets demonstrate that target users are more likely to: (1) follow the core users’ interests on diverse kinds of items and disclosure behaviors, thereby outperforming the compared methods; and (2) disclose more information with lower intrusion awareness and privacy concern.

## 1. Introduction

There has been massive growth of social media and user engagement throughout the large-scale social sensing infrastructure [1], in which people act as sensors engaging directly with the social network and exchange real-time information with each other. Specifically, the amount of information has been exponentially growing, thereby making the discovery of useful knowledge on users’ potential needs and sharing ideas more challenging, which leads to difficulty in finding and understanding valuable and truthful messages in the much larger volume of social media content. Personalization applied in sensors has attracted substantial interest in social media fields, since they can learn users’ preferences from past behaviors and interactions with items [2]. Trust-aware social sensing also utilizes users’ social networks, and the underlying assumption is that a target user’s preferences can be influenced by the decisions of their neighbors [3]. A well-known personalization method is top-*N* item recommendation, which involves suggesting a list of items that best fit users’ “like” trends [4,5]. Its basic algorithms rely on users’ purchasing, browsing, and viewing histories in real-world situations, e.g., collaborative filtering algorithms [6,7]. However, in social sensing environments, current candidate items for personalization belong to multiple kinds; therefore, adopting trust-based approaches is hard to achieve with high prediction accuracy and can raise the privacy intrusion caused by data collection, which leads to less data input to the personalization algorithm and increased user privacy concerns [8]. As a result, privacy intrusion has limited the further development of trust-aware social sensing in related research fields [9,10]; however, the possible enhancement from core users via social trust relationships provides a potential solution to this problem, which motivates our work.

In this paper, we propose a new trust-aware approach for top-*N* diverse item personalization in social sensing, which optimizes prediction accuracy while reducing users’ privacy concerns. We introduce a model, called the User and Item Similarity Model with Trust in Diverse Kinds (UISTD), which utilizes user-to-user similarity and item-to-item similarity over multiple kinds of items with common tags for higher personalization quality. With UISTD, we demonstrate that using a modified *k*-means clustering algorithm to select the core user, and their disclosure behaviors and “like” trends, can make the target users more likely to accept the predicted items and disclose more information throughout the trust relationship between the target users and the core users. In this study, we conducted experiments on three real-world data sets using both our UISTD model and other counterpart models. The UISTD model outperformed other counterparts, due to the fact that it not only applies the trust relationship and tags of various kinds of items but also eases the users’ concerns about privacy intrusion, as it provides personalization according to the core users’ preferences. The results of the application of the model to real-world data sets enabled us to determine what personalization providers in social sensing should do so that users will be more likely to accept the prediction and more willing to disclose their information with less privacy concern. Our suggestions are as follows: (1) suggest the core users’ preference on diverse kinds of items so that the target user will be more likely to accept the personalization; and (2) respect users’ privacy concerns by suggesting the core users’ disclosure pattern while collecting users’ data, so that the target users will disclose the information with lower intrusion awareness.

## 2. Related Work

This study builds on existing research on privacy intrusion and computational intelligence in social sensing, and addresses several theoretical and practical gaps in prior studies. Here, we briefly introduce related work on privacy intrusion and personalization, and describe the manner in which the problems of privacy intrusion were solved. Gaps were identified in these studies, which we subsequently discuss in this work.

There are many data- and computation-intensive applications on social sensing and embedded devices with large-scale computing infrastructure [11,12]. Users face a bewildering array of options [13,14,15,16], and many social sensing infrastructures utilize personalization for detailed user preference extraction, which can help users find the items that best fit their interests [17]. Such technology learns user preferences based on past purchasing behaviors according to browsing history and mainly applies model-based approaches, which can enhance service accuracy and be adapted to large-scale data [18]. Specifically, trust-aware social sensing personalization utilizes purchase records of not only target users but also other potentially associated users, and the user similarity is substituted by the trust relationship, which can alleviate the problem of data sparsity [19]. Many researchers use trust to enhance user similarity for better top-*N* item personalization in terms of how well the prediction matches users’ preferences. Golbeck [20] utilized trust relationship to better design interfaces and present FilmTrust to create movies that suits users’ interests. Shuiguang incorporated trust relationship in social networks with user feedback in order to optimize personalization services [21]. Guoqiang [22] suggested a novel service recommendation through users’ trust network, which achieved a good personalization performance. TRA was applied by Yingyuan to construct a new social trust matrix and fuse users’ preferences on friendship with those of other users [23]. Jian [24] proposed a media recommendation solution in heterogeneous social network called GCCR to tackle false information by a user-centric strategy. Na [25] built a trust-aware recommender system with enhanced accuracy which reliably estimates users multi-faceted and asymmetry trust strengths.

However, a newly encountered problem in trust-aware social sensing personalization is privacy intrusion; better personalization requires collecting users’ raw data at a basic level, thereby raising users’ privacy concerns. Increased privacy concerns will reduce privacy disclosure, which will lower the prediction accuracy of social sensing such that the personalization does not satisfy users’ needs at a basic level. Although the quality of a personalization improves as the volume of available personal data increases [26], users generally do not agree to this data collection [27] since there are many reports of unwanted exposure of information and illegal disclosure to third parties [28]. However, users may disclose their long-term privacy for temporary benefits, such as when filling in questionnaires in exchange for coupons and discounts. This issue, which is called the “personalization–privacy paradox”, has attracted research interest. Various researchers posit that the amount of privacy that is disclosed to a trust-aware social sensing personalization depends on how much users trust the system. Moreover, Meyffret argued that user anonymity allows users to feel less concerned about their privacy [29]. Allowing users to decide what privacy information to share is one option [30]; however, users often rely on default settings when considering their privacy controls [31]. Most users lack sufficient knowledge about personalization and privacy and are uncertain about privacy control [32]. Recent studies have proposed many methods for dealing with the problem of privacy intrusion. For example, users can be assisted with decision support for privacy disclosure. However, overcoming this problem for all users, i.e., determining how to make them less aware of privacy intrusion such that they disclose more information to facilitate personalization, remains challenging. Our work aims to better define an item ranking score for target users by incorporating their trust relationships with core users, by which their preferences are determined for rated and unrated items with common tags across diverse kinds. Using this approach, we provide a new method for designing a trust-based item ranking model with lower user awareness of privacy intrusion.

## 3. Models for Recommendation

**Figure 1:**
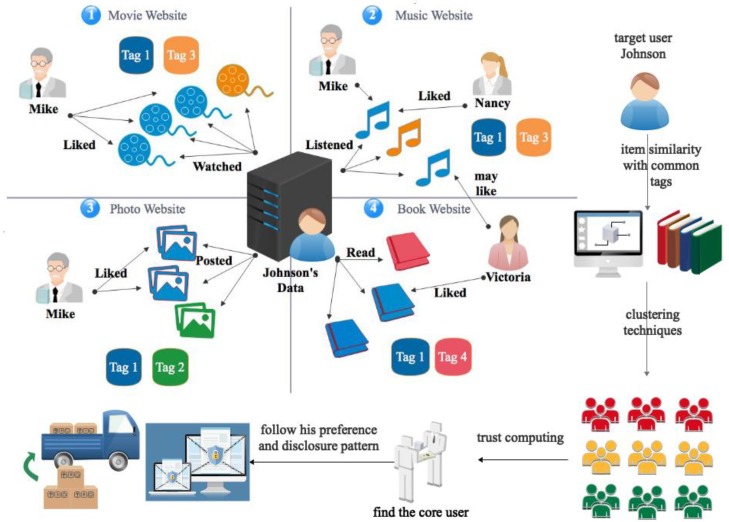
Prototype of the proposed diverse item recommendation model, namely, User and Item Similarity Model with Trust in Diverse Kinds (UISTD). UISTD first utilizes item similarity with common tags of the target user for clustering techniques, then adopts trust computing to find the core user, and finally suggests the core user’s preference and disclosure pattern to the target user.

To improve the diverse recommendation and privacy, we propose the UISTD model; its architecture is shown in Figure 1. We first introduce how other scholars apply trust in recommendation service, then how we further improve their work in the UISTD model.

### 3.1. Factored Similarity Models with Trust

This section introduces well-performing systems with decision logic and models with factored similarity, including the Factored Similarity Model (FSM)—which was proposed by Guibing et al. [33], and inspired by which we extend trust influence across various kinds of items—and Athena architectural design for decision queries proposed by Jongdeog et al. [34], to which we referred and design the personalization engine.

#### 3.1.1. Item Similarity Model

The notations used in this paper include lowercase letters to represent row vectors, e.g., *u* and *i* to denote the attributes of an user and an item. A matrix that represents a set of users or a set of items is represented by uppercase letters, e.g., *I*, etc. A predicted value, such as a ranking score for the possibility of suggesting a candidate item *i* to user *u*, is denoted by a circumflex (^) above the letter, e.g., ${\hat{r}}_{u,i}$. A ranking score ${\hat{r}}_{u,i}$ for a user *u* toward item *i* is obtained by aggregating the item similarities with other items {*j*_1_, *j*_2_,…, *j_x_*} that have already been rated by *u*. The similarity between items *i* and *j* can be expressed via two matrices, which are denoted as *X* and *Y*, where *X*, *Y* $\in {\mathbb{R}}^{m\times d}$, and d≪n denotes the number of latent features that are associated with an item. Hence, the similarity of items *i* and *j* can be obtained from $x_{j}^{T}y_{i}$, where *x_j_* and *y_j_* represent the item-specific latent feature vectors for item *i* and item *j*. The ranking score for user *u* on an unrated item *i* is calculated by:

(1) $${\hat{r}}_{u,i}=b_{i}+{\left|I_{u-i}\right|}^{-\alpha }\times \underset{j\in I_{u-i}}{\sum }x_{j}^{⊺}y_{i}$$

where *b_i_* is the bias of item *i* and *I_u−i_* = *I_u_*\{*i*} is the set of items that have already been rated by user *u*, except for the item *i*, which is currently being estimated. Matrices *X* and *Y* can be learned by recovering item ranking connections between rated item *i* and unrated item *j* for user *u* with an objective function via an item-based nearest-neighbor approach:

(2) $$J=\frac{1}{2}\underset{u}{\sum }\underset{i\in I_{u}^{+},j\in I_{u}^{-}}{\sum }\|\left(r_{u,i}-r_{u,j}\right)-{\left({\hat{r}}_{u,i}-{\hat{r}}_{u,j}\right)\|}_{F}^{2}+\frac{\lambda }{2}\left({\left\|X\right\|}_{F}^{2}+{\left\|Y\right\|}_{F}^{2}+\|b_{i,j}\|{}_{F}^{2}\right)$$

where $I_{u}^{+}$/$I_{u}^{-}$ is the set of items that user *u* has rated/not rated, ${\left\|X\right\|}_{F}$ is the Frobenius norm, and λ is a weight parameter for the regularization term, which is used to avoid over-fitting. The objective function *J* is applied to adjust the ranking scores from the aggregation of the items that have been rated by users with controlled agreement, respective to their average similarities. This model incorporates the item personalization with ranking into a matrix factorization model but fails to apply user similarity for item ranking.

#### 3.1.2. Model of Both User and Item Similarities 

Item similarity models compute a ranking score for each item. Guibing et al. proposed the FSM, which considers how strongly active users are correlated with the users who have rated the target item. Enhanced by the effects from active users’ correlations, FSM considers users who share similar tastes to be more likely to prefer the target item. The similarity of two users is computed as an inner product of two low-rank matrices, which are denoted as $P\;\mathrm{and}\;Q\;\epsilon \;{\mathbb{R}}^{m\times d}$, where *d* is the number of latent features with which a user is associated. The ranking score of user *u* for unrated item *i* is expressed as follows:

(3) $${\hat{r}}_{u,i}=b_{i}+{\left|U_{i-u}\right|}^{-\beta }\times \sum_{v\epsilon U_{i-u}}x_{v}^{⊺}y_{u}$$

where *U*_*i*−*u*_ = *U_i_*\{*u*} is the set of users who have rated item *i*, excluding user *u*; the similarity of users is computed as the inner product between users *v* and *u*, which are represented by vectors *x_v_* and *y_u_*; and β is a parameter that depends on the number of users and is restricted to the value range [0, 1] (similar to α in Equation (1)). When considering both users and items, FSM is applied with a ranking score for user *u* on item *i*, which consists of three parts: item bias *b_i_*; the similarity between user *u* and another user *v* who rated item *i*: $x_{v}^{⊺}y_{u}$; and the similarity between item *i* and another item *j* that was rated by user *u*: $x_{j}^{⊺}y_{i}$. The weight parameter *s* is applied to adjust the contribution of item similarity and user similarity, and the ranking score is expressed by the following equation:

(4) $${\hat{r}}_{u,i}=b_{i}+s{\left|U_{i-u}\right|}^{-\beta }\times \underset{v\epsilon U_{i-u}}{\sum }x_{v}^{⊺}y_{u}+\left(1-s\right){\left|I_{u-i}\right|}^{-\alpha }\times \underset{j\in I_{u-i}}{\sum }x_{j}^{⊺}y_{i}$$

#### 3.1.3. Ranking with Trust

Guibing et al. considered both item and user similarity, and especially incorporated trust to enhance the personalization performance. Active users rate items and “friend” other users who share common interests in items. Our previous work demonstrates that active users provide the major trend of what is favored by the crowd and enhance prediction accuracy. When trust is incorporated into the FSM, it is assumed that user *u* has specified a set of trusted users, which is denoted as *T_u_* = {*w*|*t*_*u*,*w*_ = 1}, and the objective is to predict a ranking score on item *i* for user *u*. When the influence of trust is incorporated into the FSM, the ranking prediction approach is expressed as follows:

(5) $$\begin{array}{ll} {\hat{r}}_{u,i}= & b_{i}+s{\left|U_{i-u}\right|}^{-\beta }\times \sum_{v\epsilon U_{i-u}}x_{v}^{⊺}y_{u}+\left(1-s\right){\left|I_{u-i}\right|}^{-\alpha }\times \sum_{j\in I_{u-i}}x_{j}^{⊺}y_{i}\\ & \;\;\;\;\;\;\;\;\;\;\;\;+{\left|T_{u}\right|}^{-\gamma }\sum_{w\in T_{u}}p_{w}^{⊺}y_{i} \end{array}$$

where α,β,γ≥0 are parameters for the numbers of rated items, similar users, and trusted users; *s* represents the relative weight of user similarity; and $p_{w}^{⊺}y_{i}$ is regarded as the amount of influence of user *w* on target item *i*. High-ranking items will be more likely to be suggested to the target users.

### 3.2. Our UISTD Model for Diverse Items

The mentioned previous works are good, however item personalization could nevertheless be further improved by considering the fact that users face a variety of item kinds on the background of social sensing. Here, we defined the prototype of the model represented in Figure 1, where a target user named Johnson has trusted friends across various social sensing media; e.g., Victoria is his friend on a book website, Nancy is his friend on a music website, and Mike is his friend on music, movie, and photo websites. Friends share favorite items of diverse types; however, they usually have common tags, which makes diverse personalization feasible. In this paper, inspired by FST [33] and Div-clustering [35], we obtain ranking scores from the viewpoint of users on various kinds of items. Items of a single type may not allow sufficient knowledge of users’ interests to be obtained, however items from diverse types can help analyze users’ potential interests by overlapping all the domains. Our UISTD model introduces notations for the detailed description of multiple kinds of information. Let $R={\left[r_{u,i,k}\right]}_{m\times n\times l}$ represent a ternary matrix of users’ behaviors (strongly dislike, dislike, not rated, like, strongly like) over items of several kinds, where $r_{u,i,k}\epsilon \left[-2,-1,0,1,2\right]$ indicates the attitude of user *u* toward item *i* of kind *k*, and *m*, *n*, and *l* refer to the number of users, the number of items, and the number of kinds of items, respectively, so the third dimension indicates the type which items belong to. Let $T={\left[t_{u,v}\right]}_{m\times m}$ represent the binary social trust network, in which *t*_*u*,*v*_ > 0 when user *u* exhibits trusting behaviors toward user *v*, e.g., *u* discloses their privacy information to *v*, including adding them as a friend, sharing their purchase history, and viewing their comments, and *t_u,v_* = 0 otherwise. The personalization objective is formulated mathematically as follows: given a set of users {*U_x_*}, past behavior ternary matrices *R* and social trust relationship matrices *T* suggest the top-*N* candidate items that the user has not viewed but is most likely to buy. The weight parameter *r* is applied in Equation (6) to adjust the contribution of item similarity, user similarity, and the kinds that items belong to on the basis of Equation (4), where parameter *s* exists. The ranking score can be computed as follows:

(6) $$\begin{array}{ll} {\hat{r}}_{u,i,k}= & b_{i,k}+s{\left|U_{i-u}\right|}^{-\beta }\times \sum_{v\epsilon U_{i-u}}x_{v}^{⊺}y_{u}+r{\left|I_{u-i}\right|}^{-\alpha }\times \sum_{j\in I_{u-i}}x_{j}^{⊺}y_{i}\\ & \;\;\;\;\;\;\;\;\;\;\;+\left(1-s-r\right){\left|T_{u,v}\right|}^{-\gamma }\sum_{w\in T_{u}}d_{u}^{⊺}y_{i} \end{array}$$

where *b_i,k_* represents the bias of user *u* on item *i* of kind *k*, and *s*, *r*, *r* + *s* ∈[0,1] stand for the relative importance of user similarity and their trust relationship. For users who disclose privacy to each other, $d_{u}^{⊺}y_{i}$ is regarded as the number of disclosures from user *u* on the target item, namely, item *i*.

#### 3.2.1. Basic Data Structure

The data structures of users and items that are loaded onto the model are as follows:

**User (Id, Profile, Friends [Id_1_, Id_2_,…, Id*_x_*], ViewHistory, Kinds [*k*_1_, *k*_2_, …, *k_y_*])**

A user may use different names on different sites to which he or she has registered. However, the model should only use one id to identify each user. This data structure also includes private user information, e.g., age, gender, phone number, post code, and affiliation, which can be collected from all the sites under the users’ permissions and should be stored in the user profile. A user can add other users as their friends in the list of *Friends*, while *ViewHistory* records users’ browsing histories for all types of items in the following format:

**Item (Id, Category, Tags [*t*_1_, *t*_2_,…, *t_i_*], Weight, Website)**

An item must be associated with a unique id, while *Category* records the groups to which this item belongs, e.g., books, flowers, and stories. When an item is uploaded by a user to a website for the first time, the user can add descriptions of this item with several tags, e.g., romance, comedy, and tragedy. *Tag* indicates the kind to which this item belongs. Although items may be listed on different websites, they can be correlated with each other. Users can rate an item on a scale of 1 to 5, and the average rating is calculated as the *Weight*.

#### 3.2.2. Personalization Engine

UISTD is equipped with a personalization algorithm that calculates the recommended items in three steps: First, the algorithm determines the friends and computes their similarities on real-time decisions [35]. Specifically, the similarities among friends are given by the common “ratings”, and *S_friends_* represents how many same ratings two users have in common, as defined by Pearson’s correlation coefficient:

(7) $$S_{friends}=\frac{Weight_{x,y}}{\sqrt{Weight_{x}^{2}\times Weight_{y}^{2}}}$$

where *Weight* represents the overlapping times that two users give same comments on several items. Specifically, the parameter *Weight*_*x*,*y*_ is the total *Weight* of the rated items that users *x* and *y* have in common, while *Weight_x_* is the total weight of the similar items that are rated by user *x*. The *Weight* of each item is initialized to 1. Recommendations for user *u* are then generated by assigning *Weights* to item *i* of kind *k* according to ratings by friends:

(8) $$Weight=\underset{S_{friends}}{\sum }{\hat{r}}_{u,i,k}$$

where the *Weight* of recommended item *i* is the sum of the ratings that are given by friends. All top-ranking recommended items are presented to users in decreasing order of *Weight*. Finally, items that are rated by the users are removed and the item of highest priority that has common tag(s) with the removed items is recommend.

#### 3.2.3. Clustering and Core User

UISTD will inform users about what their friends are doing on all sites and generate personalization for items by calculating the possibility that a user may like each item. UISTD investigates whether target users will follow core users’ disclosure behaviors when the target users come upon a site with which they are not familiar. The privacy disclosure solution for a target user is valid only when: (1) they disclose their privacy at the same level as the core users; and (2) they accept the predicted top-ranking items from UISTD. Before applying the privacy disclosure solution, UISTD clusters the users such that all users will be suggested to follow the closest core user. The *k*-means clustering approach has been modified as follows to cluster the users.

Suppose that the whole group of users is a set of *m* entities, which are denoted as (*E*_1_, *E*_2_,…, *E_m_*). Each entity *E_x_* is expressed as *E_x_* (*R_x_*_1_, *R_x_*_2_, *R_x_*_3_,…, *R_xn_*), where *R_xj_* (*j* = 1, 2,.., *n*) stands for a recorded rating from user *x* on item *j*. The entities are divided into clusters according to their rating records, and each cluster regards a core user as its center. An entity is included in a cluster if it has properties that are most similar to those of its core user, over the core users of all clusters. The entities have trust relationships with each other, and the relationships are represented by *m* − 1 edges; hence, the total number of edges is T=m×(m−1)/2. The distance $D=\sum_{u=1}^{m}\sum_{v\neq u}\left(E_{u},E_{v}\right)/2$ measures the gaps between entities, which corresponds to how unlikely they are to disclose information to each other, where *m* is the total number of entities and the routine (*E_u_*, *E_v_*) stands for the rating differences between *E_i_* and *E_j_*:

(9) $$rountine\left(E_{u},E_{v}\right)=\sqrt{{\left({\hat{r}}_{u,1,k}-{\hat{r}}_{v,1,k}\right)}^{2}+{\left({\hat{r}}_{u,2,k}-{\hat{r}}_{u,2,k}\right)}^{2}+\ldots +{\left({\hat{r}}_{u,n,k}-{\hat{r}}_{u,n,k}\right)}^{2}}$$

where *k* is the kind that item *i* (*i* = 1, 2,.., *n*) belongs to. Users apply the same *tag* to items of the same *Kind*. An entity *E_x_* is regarded as abnormal if routine (*E_x_*, *E_y_*) > *D*/*T*, because this indicates that their ratings are too far away from those of other users.

We denote the number of clusters by *k*. For *m* users, *k* = 1 when all users are in the same cluster and *k* = *m* when each user belongs to their own cluster. Hence, the value of *k* varies from 1 to *m*. Another parameter, namely, the minimum distance variance in *n* folds, which is denoted as *MDV_n_*, is introduced. Users’ data sets are randomly divided into 10 folds, which are numbered from 1 to 10. For each turn, one fold is regarded as a testing set, while the remaining nine folds are applied as a training set and *n* is the fold number:

(10) $$MDV_{n}=\overset{k}{\underset{j=1}{\sum }}\underset{x\epsilon C_{j}}{\sum }routine\left(m,U\right)$$

where *k* is the total number of clusters, *x* is the user set in cluster *C_j_*, and *m* is the core user. Each time the value of *k* changes, *MDV_n_* is computed accordingly. The minimum value of *MDV_n_* is recorded and the corresponding value of *k* is recorded as *k_n_*, which is regarded as the number of clusters. The corresponding value of *k_n_* is the final value.

#### 3.2.4. Privacy Disclosure Solution

**Figure 2:**
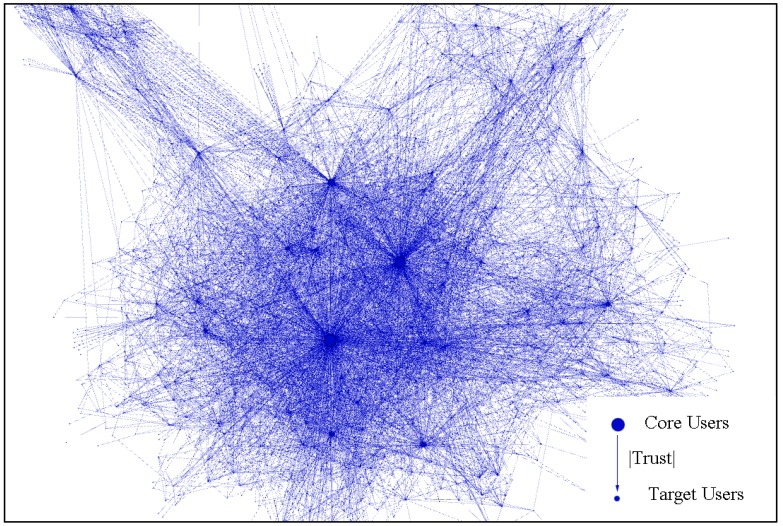
Users’ trust relationships are modeled as a network; the disclosure volume between users indicates how close they are and the value of *Trust* distinguishes the core users and other target users.

When target users are confronted with privacy threats, e.g., intrusion detection, it is assumed that they will not only tend to accept the core users’ like trends on items but also follow the core users’ disclosure decisions. This was confirmed in our previous work [36]. We extend the solution to disclosure patterns among trusted relationships. An edge can be weighted according to the number of disclosure behaviors that have occurred and the total number of disclosures, which represents how tight the trust relationship is between a target user and a core user. A trust relationship can take many forms. For instance, a user might have talked with someone and found that they share a common interest, and hence they entered into a trust relationship. Trust relationships can also be formed via introductions through other trust relationships on a site. If a user *u* has an edge connection with another user *v*, they will form a trust relationship, which indicates the possibility of information disclosure. Trust relationships can be divided into three levels depending on how many times disclosures have occurred and the total amount of information that has been disclosed. As shown in Figure 2, each node represents a user and its size indicates how many close friends this user has. The biggest node stands for a core user, who is trusted by the most users. The maximum disclosure volume may represent the amount of information that is disclosed in a trust relationship between close friends, while the lowest volume represents the amount of information that is disclosed with a stranger.

Here, we formally define Trust and Disclosure:

**Trust from user *u* (User *u*’, String cluster, float *strength score*)**

Each user *u* maintains a trust list that specifies who can be a disclosure receiver. This contains a set *U_u_*’ of all users, such as *u*’, that have a trust relationship with *u*. A cluster represents the level of friendship with *u*’, e.g., high, normal, or low. The *strength score* indicates the level of proximity to user *u*’ in the same group, where a higher score represents a closer relationship with user *u*, to whom these users may disclose more privacy. When the sensitivities of two disclosures *i* and *i*’ are the same, they can be placed into the same cluster. When a user *u* can disclose content *i*, *u* may also disclose content *i*’ if *i* and *i*’ belong to the same cluster. Our implemented definition of Disclosure is as follows:

**Disclosure** ***d* (Cluster *c*, float *sensitivity score*)**

For each Disclosure *d*, similar items can be determined, such as *d*’, according to their clusters. The *sensitivity score* quantifies the similarity between Disclosures *d* and *d*’, where a higher score indicates that the users are less likely to disclose information. The disclosure pattern will be predicted based on the Disclosures {*D*} and privacy receivers {*U*} whose clusters contain the same levels of Trust and Disclosure. For example, we found that a core user *u_c_* disclosed content *d* of Trust type normal and predict that another user u’ (u’ and u are in a same cluster c) will also disclose d’ (d’ and d are both normal). In the experiment section, we will evaluate the accuracy of the predicted disclosure pattern.

## 4. Experiments

### 4.1. Data Sets

Three real-world data sets are used in this section, including Epinions and Ciao, which are comprised of users’ reviews and numerical ratings of a variety of products. The third data set comes from our previous work, in which we hired users who had at least five accounts on social sensing websites, through a crowdsourcing platform, namely, Sojump, which is a website that provides online survey services and connects more than two million individuals and businesses to coordinate the use of human intelligence. Previously supported data “DLPDS” were used to support this study and are available at relevant place within the text as reference [36]. All three data sets include both user-item ratings and user-to-user trust relationships. To prevent sparsity problems, we remove the lowest 20% of users in terms of the number of ratings they had given. The specifications of the three data sets are listed in Table 1. All rating values were preprocessed to binary values (0 or 1), which indicate whether or not a user has rated an item.

### 4.2. Evaluation Metrics

The 10-fold cross-validation approach was adopted; for each iteration, nine subsets were used as the training set and the remaining subset was used as the testing set. The lowest *MDV_n_* over 10 execution results is reported as the final performance. We adopted two popular ranking metrics to evaluate personalization performance, namely, precision and F1-measure at N (*P@N* and *F1@N*), where the cutoff N is chosen in {5, 10}, which represents the number of recommended items:

(11) P@N=1|U′|∑u∈U′|RN(u)∩I′u|N)

(12) R@N=1|U′|∑u∈U′|RN(u)∩I′u||I′u|

(13) $$F1@N=\frac{2\times P@N\times R@N}{P@N+R@N}$$

where ${I^{\prime }}_{u}$ is the set of items that are rated by user *u*, *U*’ is the set of users in the testing set, and *R@N* denotes the measurement of recall at N. The F1-measure represents a trade-off between ranking precision and recall. Higher values of *P@N* and *F1@N* demonstrate better top-*N* item performance. As suggested by Guibing et al. [33], we tune the values of parameters α,β and γ in {0.5, 1, 2} and *s* and *r* in [0.1, 0.3] in Equation (1). Parameter ρ is fixed as 10, as suggested by Kabbur et al. [37].

### 4.3. Comparison with Other Methods

The most basic non-personalized approach, namely, MostPop, by Zhao et al. [38], is regarded as the baseline that computes the ranking score of each item by its popularity among users and comments, i.e., how many good comments users have provided on it. FISM was proposed by Kabbur et al. [37], and adopts item similarity for ranking score of personalized item by Equations (1) and (2). FSM was proposed by Guobing et al., and utilizes item similarity and active user similarity with trust computing in Equations (3)–(5) to calculate ranking score. Our UISTD model uses Equation (6), and further extends the above algorithms by item kinds. Table 2 presents the personalization performances of all comparison methods across the three data sets in terms of P@N and F1@N, respectively. The best performance among the methods and that of our approach are shown in bold text for comparison. Generally, our approach (UISTD) obtains the best performance among all methods.

We have also added more comments of the data set results. MostPop provides most basic personalized items which computed from the popular items of users’ interests, so we believe it can be regarded as the baseline method. FISM has added item similarity in computing the personalized items. The performance of FISM outperforms that of MostPop. Furthermore, FSM extends FISM by computing both item similarity and user similarity of active users on the trust network. As a result, the precision (e.g., 0.129 > 0.1147 on Epinions, 0.2916 > 0.2704 on Ciao, 0.3318 > 0.3018 on DLPDS) and F1-measures (0.1396 > 0.1307 on Epinion, 0.2614 > 0.2495 on Ciao) are generally higher with FSM than FISM. Social trust is noted to impose important influence on the ranking performance, and users are more likely to accept the personalized items from active users’ preferences, e.g., herding effect. Finally, UISTD aggregates the knowledge trained from the item similarity and user similarity across multiple item kinds, and the overlapping interests of the users on joint domain has made up for the sparsity problem from single type of items. Meanwhile, the DLPDS data set has more dense data with more trust relationships; therefore, UISTD performed best on this data set. This demonstrates that by gaining enough trust connections from all user networks we can better identify the users’ interests and enhance the prediction accuracy. All tests were run on a server with 32 Genuine Intel(R) CPUs (2.6 GHz) and 2 GB memory. For each execution, the average time for UISTD on DLPDS, Ciao, and Epinions was approximately 7, 23, and 53 min, respectively.

Since predicted items in UISTD are generated via a modified *k*-means clustering algorithm, we assume that all target users follow the disclosure behaviors of their core users, who belong to the same cluster. In this part, we only consider the performance of UISTD on DLPDS, where 717 participants from Sojump with unique IP addresses responded to our study, 693 of whom were qualified for further analysis; the others were disqualified since they provide false information. Each user was randomly assigned to one of two scenarios: In Scenario 1 (n = 351), users were informed of what the core users had disclosed and what was being “liked” by the core users, to facilitate personalization. In Scenario 2 (n = 342), users were only informed of what was being liked by the core users. We mainly examined the tension between the disclosure volume and the prediction accuracy. The privacy disclosure includes personal information requests (e.g., address location, gender, and commuting route) and online information requests (e.g., favorite movie and homepage on browser).

**Figure 3:**
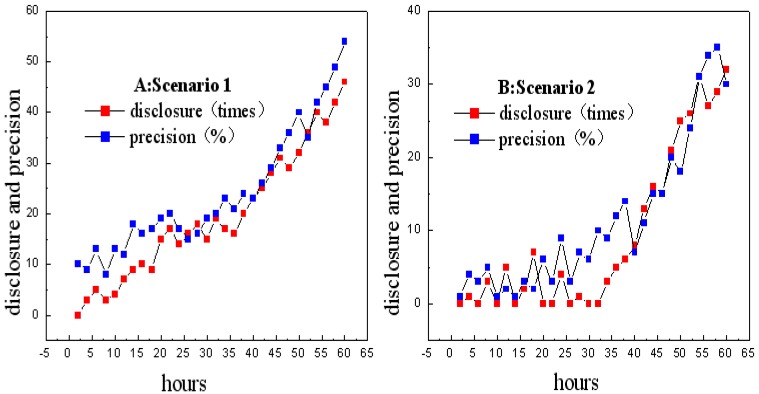
Information disclosure volume and prediction accuracy in Scenarios 1 and 2.

The process was run for 60 h. As shown in Figure 3, users in Scenario 1 shared more information (average disclosures = 16; Figure 3A) than the users in Scenario 2 (average disclosures = 5; Figure 3B), especially in the first 30 h. Meanwhile, the prediction accuracy for predicting the items was comparatively higher for UISTD in Scenario 1 (average prediction accuracy = 27.61%) than in Scenario 2 (average prediction accuracy = 11.67%). We believe the higher amount of collected information increased the prediction accuracy. In the last 30 h, users demonstrated higher disclosure volume (average disclosures 46 > 19) and the personalization engine obtained higher prediction accuracy (average 39.43% > 23.71%).

**Figure 4:**
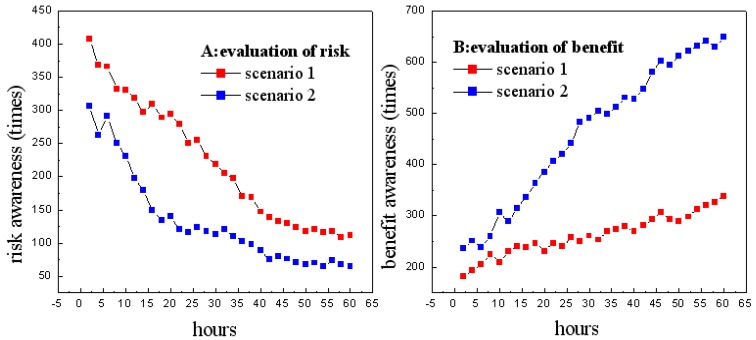
Users’ evaluations of potential risk (**A**) and potential benefit (**B**).

We require the users to forward their comments during their participation in the experiment in both scenarios. The comments can be positive, e.g., “I believe the privacy requesting strategy is good”, or negative, e.g., “The information requesting is terrible and offensive”. We use natural language-processing techniques to semantically determine whether each comment is positive, negative, or neutral. Two counters, which are denoted as *P* and *N*, are initialized to 0 to record the numbers of positive and negative comments. Whenever a positive comment is identified, the counter *P*.counter is incremented by 1, and whenever a negative comment is identified, the counter *N*.counter is incremented by 1. We regard the negative comments as users’ awareness of potential risks. As shown in Figure 4A, users’ risk awareness generally decreases in both scenarios, which demonstrates that the personalization engine provides high-quality suggestions of what items to view and helps gain users’ trust via the personalization benefits. Comparatively, in Scenario 1, which has a low rate of negative comments, UISTD successfully mitigated the privacy problem. In contrast, in Scenario 2, UISTD encountered the problems of cold start and user awareness of privacy intrusion, which may be caused by the privacy collection strategy, which does not consider core users.

## 5. Conclusions and Future Work

This article proposed a model, namely, UISTD, that incorporates trust relationships for the top-ranking of diverse item personalization in social sensing infrastructure. Both item-to-item similarity and user-to-user similarity were applied in a matrix factorization algorithm, and diverse kinds of items were also considered. UISTD utilizes a modified *k*-means clustering algorithm to select the core users, and their disclosure behaviors and like trends can lead the target users to be more likely to accept the personalization and disclose more information, via the trust relationships between the target users and the core users. We conducted experiments on three real-world data sets, and demonstrated that our UISTD model outperformed its counterparts in terms of prediction accuracy of personalization and disclosure volume, as it not only applies the trust relationships and tags of various kinds of items but also reduces the users’ awareness of privacy intrusion. This statement is supported by semantic evaluation of users’ comments on potential risk vs. benefits, and the UISTD model performs best. This paper considered users’ privacy concerns regarding data collection in the personalization of social sensing; however, it did not analyze how users’ features will affect how much they feel intruded upon. In future work, we plan to incorporate users’ features into a personalized privacy collection strategy and investigate the motivations behind users’ privacy disclosure intentions related to social sensing. Hopefully, additional interesting phenomena will be identified.

## Figures and Tables

**Table 1:** Specifications of the data sets, where Density = |Ratings|/(|Users|×|Items|×|Trusts|).

Data Set	|Users|	|Items|	|Ratings|	|Trusts|	Density
Epinions	31,922	139,738	51,093	42,109	0.03%
Ciao	5970	99,746	25,108	89,219	0.13%
DLPDS	553	872	10,739	760	4.13%

**Table 2:** Experimental results on three data sets in terms of precision and F1-measure. The best performance among the approaches is highlighted in bold to facilitate comparison.

Measurement	Precision				F1-Measure			
Data Set	MostPop	FISM	FST	UISTD	MostPop	FISM	FST	UISTD
Epinions	0.1169	0.1147	**0.1290**	**0.1290**	0.1298	0.1307	**0.1396**	**0.1417**
Ciao	0.2677	0.2704	**0.2916**	**0.3017**	0.2436	0.2495	**0.2614**	**0.2756**
DLPDS	0.2915	0.3018	**0.3318**	**0.3812**	0.3032	**0.4216**	0.3717	**0.4218**
